# Bidirectional Coupling between Astrocytes and Neurons Mediates Learning and Dynamic Coordination in the Brain: A Multiple Modeling Approach

**DOI:** 10.1371/journal.pone.0029445

**Published:** 2011-12-29

**Authors:** John J. Wade, Liam J. McDaid, Jim Harkin, Vincenzo Crunelli, J. A. Scott Kelso

**Affiliations:** 1 Intelligent Systems Research Centre, School of Computing and Intelligent Systems, University of Ulster, Derry, Northern Ireland; 2 Neuroscience Division, Cardiff School of Biosciences, University of Cardiff, Cardiff, United Kingdom; 3 Center for Complex Systems and Brain Sciences, Florida Atlantic University, Boca Raton, Florida, United States of America; Georgia State University, United States of America

## Abstract

In recent years research suggests that astrocyte networks, in addition to nutrient and waste processing functions, regulate both structural and synaptic plasticity. To understand the biological mechanisms that underpin such plasticity requires the development of cell level models that capture the mutual interaction between astrocytes and neurons. This paper presents a detailed model of bidirectional signaling between astrocytes and neurons (the astrocyte-neuron model or AN model) which yields new insights into the computational role of astrocyte-neuronal coupling. From a set of modeling studies we demonstrate two significant findings. Firstly, that spatial signaling via astrocytes can relay a “learning signal” to remote synaptic sites. Results show that slow inward currents cause synchronized postsynaptic activity in remote neurons and subsequently allow Spike-Timing-Dependent Plasticity based learning to occur at the associated synapses. Secondly, that bidirectional communication between neurons and astrocytes underpins dynamic coordination between neuron clusters. Although our composite AN model is presently applied to simplified neural structures and limited to coordination between localized neurons, the principle (which embodies structural, functional and dynamic complexity), and the modeling strategy may be extended to coordination among remote neuron clusters.

## Introduction

For many years, astrocytes, a subgroup of glial cells found in the brain, have been thought to support neurons by providing them with vital elements needed for their survival [1]–[3]. In recent years, several new discoveries have revealed that astrocytes can encapsulate ∼10^5^ synapses and can connect to multiple neighboring neurons [4], [5]. Although astrocytes cannot elicit propagating action potentials (APs) like neurons do, they can communicate in a bidirectional manner with neurons and other astrocytes by release of transmitters (which include glutamate and adenosine triphosphate (ATP) referred to as gliotransmitters) and propagating calcium (Ca^2+^) waves. In particular, the interaction of glutamate with astrocytic receptors leads to transient elevation in astrocytic intracellular Ca^2+^ levels [6]–[9], which represent a fundamental mode of excitation in astrocytes. In response to these Ca^2+^ transients, astrocytes release gliotransmitters which in turn modulate synaptic transmission by acting both on pre- and post-synaptic receptors. As well as intracellular communication, astrocytes communicate with each other through the propagation of Ca^2+^ waves, a process which is thought to be mediated via extracellular ATP diffusion and the transmission of inosotil 1, 4, 5-trisphosphate (IP_3_) through gap junctions. However, the exact nature of this process is still unclear [10]–[14].

Traditionally, communication and information transfer within the brain have been the sole province of pre- and post-synaptic coupling between neurons. However, recent research has extended if not challenged this view of synaptic physiology. The coupling of astrocytes and neurons results in an intimate connection which provides a pathway for chemical communication between the cells: a synapse actually exchanges signals at three terminals, hence the name *tripartite synapse* [15]. Neuron to astrocyte communication is promoted by glutamate which is released across the synaptic cleft upon arrival of a presynaptic AP. Some of the released glutamate binds to metabotropic glutamate receptors (mGluRs) of the connected astrocyte resulting in an astrocytic intracellular release of IP_3_. This in turn regulates the release of Ca^2+^ from internal stores, creating a transient increase in Ca^2+^ (for a detailed review see [10], [16]). Moreover, the intracellular Ca^2+^ increase has also been shown to propagate intracellularly in a process which is believed to be promoted by the propagation of signaling proteins between neighboring microdomain clusters of IP_3_ receptors [17], [18].

Astrocytes also communicate in a feedback mode with neurons and have been found to play key roles in Long Term Potentiation/Depression (LTP/LTD) [19], [20] and neuronal synchrony [21]. In response to elevated levels of Ca^2+^, gliotransmitters such as glutamate are released, leading to activation of extrasynaptic glutamate receptors (NR2B subunits of N-methyl-D-asparate receptors or NMDARs) on the postsynaptic neuron, mainly located at dendritic spines [22]. This NMDAR-activation brings about the characteristic hallmark of astrocyte-neuron signaling, i.e. a slow inward current (SIC), which has a rise time of ∼60 ms and a decay time of ∼600 ms, and is thus very different from the classical excitatory postsynaptic current (EPSC) (∼6.4 ms and ∼10 ms rise and decay time respectively) elicited by glutamate released from the presynaptic neuron [21], [23]. As well as producing local SICs, it has been found that glutamate release also acts on neighboring neurons and produces synchronized SICs [21]. Astrocytes can also release glutamate spontaneously in the absence of synaptic activity [24] supporting a role for astrocytic glutamate release in the synchronization of neighboring neurons [25], [26]. One explanation for synchrony is that neighboring neurons can sense astrocyte related glutamate release in the extracellular space. Another hypothesis is that a pair of synchronized releases occurs from two distinct sites of the same or different astrocytes [21]. Since an astrocyte interacts directly with an average of six neurons and can associate with between 300 and 600 dendrites with no overlap of astrocyte territories, it is unlikely that synchronization is due to different astrocytes connected to neurons. A more likely explanation is that neighboring synapses are coordinated by signals from a single astrocyte [4], [5]. Furthermore, the degree of synchrony precision rules out the spread of Ca^2+^ waves throughout a glial network [26]. It is interesting to note that the astrocyte-elicited SICs are often much larger than synaptic NMDA currents, i.e. ∼100 pA [23], [26], and therefore are an ideal candidate for the synchronization of neural activity.

In this paper, we model neuron-astrocyte interaction and provide evidence which shows that astrocytes have a role to play in LTP/LTD where neuron-astrocyte interactions at a synaptic site can cause plasticity at other remote sites via SICs. Also we show how an astrocytic induced signal can cause dynamic synchronization between neurons. Much evidence indicates that cognitive and behavioral functions rely on flexible coordination among distributed neural activities within and between cortical areas (see [27], [28] for reviews). However, although several mechanisms have been proposed for synchronization, its physical basis remains obscure. Our model shows how dynamic coordination in the brain may emerge from bidirectional communication between neurons and astrocytes.

## Materials and Methods

A feature of the present modeling approach is its constructive nature: it combines and constructs multiple detailed models in order to reveal the regulatory dynamics of astrocytes at a network level. In this it contrasts to larger-scale network approaches in which statistical descriptions of neural populations allow one to derive generic differential equations that describe the evolution of the averaged activity of pools of equivalent neurons (see [29] for review). The present work builds on two biophysically motivated models which describe the interactions between astrocytes and neurons in a tripartite synapse: the gatekeeper model [30] and the Nadkarni and Jung model [31], [32]. Both of these models use Li and Rinzel Ca^2+^ dynamics [33] to describe the evolution of synaptically driven Ca^2+^ transients in the astrocyte, which in turn modulates synaptic transmission via the release of astrocytic glutamate that binds to presynaptic receptors. However, no attempt has been made to investigate how the binding of astrocytic glutamate to the postsynaptic neuron affects long/short term synaptic coupling. Our extended Astrocyte Neuron (AN) model uses astrocyte-driven SICs (i.e. extrasynaptic NMDA NR2B mediated neuronal currents) to provide a teaching signal for learning and to synchronize neural activity between neurons.

### Astrocyte – Neuron Interactions

**Figure 1:**
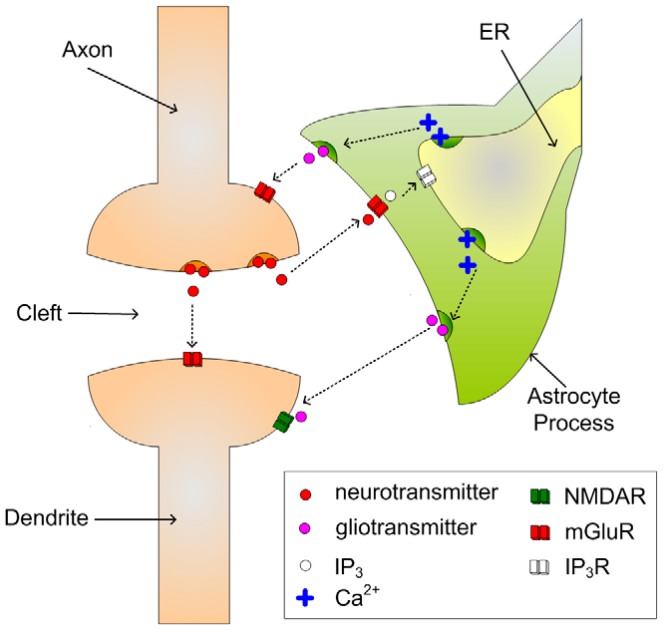
A Tripartite Synapse. The axon and dendrite, which are involved with the release and postsynaptic action of neurotransmitter respectively, are also connected to an astrocyte process which is sensitive to neurotransmitter. In response to neuronal neurotransmitter release the astrocyte can release further neurotransmitter (called gliotransmitter) which regulates the Excitatory Post-Synaptic Current (EPSC) generated by the postsynaptic neuron.

Both the gatekeeper [30] and Nadkarni and Jung [31], [32] models describe the interaction of astrocytes and neurons via the tripartite synapse. In a tripartite synapse an astrocyte process connects with the axon and dendrite of the pre- and post-synaptic neurons and is sensitive to the neurotransmitters within the extracellular fluid in the synaptic cleft [15]. Figure 1 illustrates a tripartite synapse. When neurotransmitter, e.g. glutamate, is released into the synaptic cleft by the presynaptic terminal, some of it interacts with glutamate receptors (mostly mGluRs) on the astrocyte. This then initiates the creation and release of IP_3_ into the astrocytic cytoplasm. IP_3_ subsequently binds to IP_3_ receptors (IP_3_Rs) on the Endoplasmic Reticulum (ER), a long network of tubes and vesicles used to store Ca^2+^ within the cell [34]. The binding of IP_3_ with IP_3_Rs opens channels that allow the release of Ca^2+^ from the ER in to the cytoplasm (so-called Ca^2+^ puff). Whereas individual Ca^2+^ puffs are incapable of propagating intracellularly, several puffs can raise Ca^2+^ levels in the cytoplasm beyond a threshold (believed to be of the order 0.2–0.4 µM [35]) and an oscillating Calcium Induced Calcium Release (CICR) propagation may be observed [36]. The increase in cytosolic Ca^2+^ then causes the release of transmitter, more commonly called gliotransmitter, back into the synaptic cleft. Therefore, the astrocyte can modulate synaptic transmission between pre- and post-synaptic neurons based on the previous activity of the synapse and the type of inhibitory or excitatory transmitter released.

The binding of glutamate to their related receptors on the astrocyte process and generation/evolution of IP_3_ within the gatekeeper model [30] is assumed to be dependent on the amount of neurotransmitter released, and is given by:
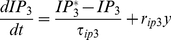
(1)where *IP_3_* is the amount of *IP_3_* in the cytoplasm. 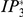 is the baseline level of *IP_3_* within the cytoplasm when the cell is in a steady state and receiving no input, *r_ip3_* is the rate at which *IP_3_* is produced, *τ_ip3_* is the *IP_3_* decay rate and *y* is the amount of neurotransmitter released into the cleft (as later described in equations (12–14)). From equation (1) it is clear that IP_3_ levels will be maintained as long as there is an input stimulus to the synapse. Furthermore, IP_3_ levels will reach a steady state based on the maintained input stimulus frequency, i.e. the higher the input stimulus frequency, the higher the level of IP_3_ (see Figure S1).

### Astrocyte Ca^2+^ Dynamics

To describe the Ca^2+^ dynamics within an astrocyte, the gatekeeper [30] and the Nadkarni and Jung models [31], [32] employed the Li-Rinzel model [33]. Although a number of computational models may describe cellular Ca^2+^ dynamics (see [37], [38] for review), it has been shown that the Li-Rinzel model can exhibit Amplitude Modulation (AM) and Frequency Modulation (FM) encoding of the cellular IP_3_ levels, as well as a mixture of AM and FM (AM-FM), via the adjustment of different model parameters [39]. Therefore we use the Li-Rinzel model to explore the effects of different encoding schemes on astrocyte-neural communication.

Ca^2+^ dynamics within the Li-Rinzel model are described by three channels. *J_pump_* which models how Ca^2+^ is stored within the ER by pumping Ca^2+^ out of the cytoplasm into the ER via Sarco-Endoplasmic-Reticulum Ca^2+^-ATPase (SERCA) pumps, *J_leak_* which describes the amount of Ca^2+^ released by leakage through the ER membrane and *J_chan_* which models the opening of Ca^2+^ channels by the mutual gating of Ca^2+^ and IP_3_ concentrations. Since the model only considers the case of a single cell which exists in a Ca^2+^-free extracellular environment, no account is taken of any Ca^2+^ flux across the cell membrane [40]. The Li-Rinzel model is described using the following equations (a full derivation of these equations is provided in [39]):
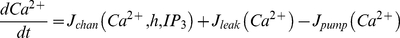
(2)

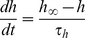
(3)where *J_chan_* is the *IP_3_* and *Ca^2+^* dependent Ca^2+^ release, *J_pump_* is the amount of Ca^2+^ pumped from the cytoplasm into the ER via the SERCA pumps, *J_leak_* is the Ca^2+^ which leaks out of the ER and *h* is considered to be the fraction of activated IP_3_Rs. The parameters 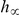 and 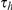 are given by:
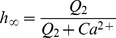
(4)and
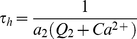
(5)where
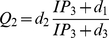
(6)The description of the *J_chan_* channel is given by:
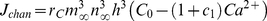
(7)where *r_C_* is the maximal CICR rate, *C_0_* is the total free Ca^2+^ cytosolic concentration, *C_1_* is the ER/cytoplasm volume ratio and *m_∞_* and *n_∞_* are the IP_3_ Induced Calcium Release (IICR) and CICR channels respectively and are given by:
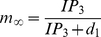
(8)and
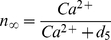
(9)The remaining channels are given by:
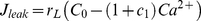
(10)and
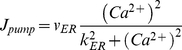
(11)where *r_L_* is the Ca^2+^ leakage rate, *v_ER_* is the maximum SERCA pump uptake rate and *k_ER_* is the SERCA pump activation constant. Table 1 provides a full description of all parameters. Note that by adjusting *C_0_* and *k_ER_* as described by [39], it is possible for the AN model to operate in three different modes i.e. AM, FM and AM-FM.

**Table 1:** Astrocyte Parameters.

Astrocyte Parameter	Parameter Description	Value
IP^*^_3_	Baseline value of IP_3_	*0.16 µM*
*r_IP3_*	rate of IP3 production	*7.2 µM s^−1^*
*τ_IP3_*	IP3 degradation time constant	*7 s*
*τ_Ca_*	Decay rate of f controlled by level of Cytosolic Ca^2+^	*4 s*
*r_C_*	Maximum rate of CICR	*6 s^−1^*
*r_L_*	Ca2+ leakage rate from ER	*0.11 s^−1^*
*v_ER_*	Maximum rate of SERCA uptake	*0.9 µM s^−1^*
*c_0_*	Total free Ca^2+^ cytosol concentration	*AM, AM-FM = 2 µM* *FM = 4 µM*
*k_ER_*	SERCA pump activation constant	*AM = 0.1 µM* *AM, AM-FM = 0.051 µM*
*c_1_*	Ratio of ER volume to cytosol volume	*0.185*
*d_1_*	IP3 dissociation constant	*0.13 µM*
*d_2_*	Ca2+ inactivation dissociation constant	*1.049 µM*
*d_3_*	IP3 dissociation constant	*0.9434 µM*
*d_5_*	Ca2+ activation dissociation constant	*0.08234 µM*
*a_2_*	IP3R Ca2+ inactivation binding rate.	*0.2 µM s^−1^*

Note: these parameters are taken from [30], [39]. All parameter values are for AM mode unless otherwise stated.

### Synapse model and astrocyte feedback

Synaptic information transfer is considered to be probabilistic: on arrival of an AP a vesicle is either released or it is not [41]. However, modeling a synapse in this way requires an extreme amount of computational time because of the probabilistic nature of synaptic transmission. Although many probabilistic models exist for the release mechanism of synapses [42]–[45], here we use a deterministic dynamic synapse model developed by Tsodyks et al. [46]. The evolving state of the synapse in this model is described by:
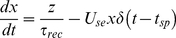
(12)

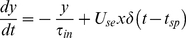
(13)

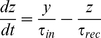
(14)where *x*, *y*, and *z* are the fractions of resources in the recovered, active, and inactive states of the synapse respectively, *τ_rec_* and *τ_in_* are the recovery and inactive state time constants respectively, *t_sp_* is the time series of presynaptic action potentials, *δ* is the Dirac delta function and *U_se_* is the utilization of synaptic efficacy. In the case of an excitatory synapse, which releases glutamate, these variables can be equated to the dynamics of glutamate release; *y* can be considered to be the amount of released glutamate while *x* can be equated to the amount of glutamate stored in the presynaptic vesicle pool ready for transmission. The EPSC that is received by a neuron from synapse *i* is proportional to the fraction of resources remaining in the active state and is given by:
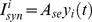
(15)where 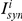 is the current supplied to the neuron from synapse *i*, *A_se_* is the absolute synaptic efficacy and *y_i_(t)* is the amount of neurotransmitter released by the synapse at time *t*.

Astrocytes influence synaptic transmission/modulation based on levels of intracellular Ca^2+^. Rather than describe this mechanism in a biophysical manner, the gatekeeper model [30] uses a phenomenological gating variable *f*. When Ca^2+^ levels within the cytoplasm exceed a set threshold (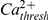), the astrocyte process releases a finite amount of gliotransmitter (glutamate) into the cleft which binds to presynaptic receptors; in doing so the transmission properties of the synapse may be changed based on astrocytic feedback. The gating variable *f* is given by:
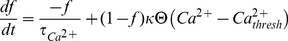
(16)where 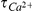 is the Ca^2+^ time constant, κ is a constant, 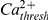 is the Ca^2+^ threshold value, Θ denotes the Heaviside function and 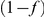 is a saturation term that reflects the fact that astrocytes have a limited amount of neurotransmitter (see [47] for detailed review of exocytosis by astrocytes). As *f* results in the release of excitatory glutamate, we would expect this to strengthen the ESPC in the postsynaptic neuron. However, it has been shown that astrocyte synaptic stimulation reduces the size of EPSCs and Inhibitory Post-Synaptic Currents (IPSCs) [48]. This is due to the fact that presynaptic mGluRs regulate the presynaptic Ca^2+^ channels which in turn reduces the flux of Ca^2+^ during incoming spike trains, therefore reducing the amplitude of EPSCs. The gatekeeper model [30] used this phenomenon as the main role of *f*, subsequently controlling the amount of neurotransmitter released into the cleft during activity. To model the effects of the glutamate released by the astrocyte in the tripartite synapse the following modifications to equations (12) and (13) are made:
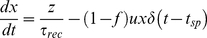
(17)

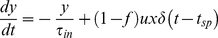
(18)From equation 18 we can see that the amount of neurotransmitter release across the cleft is proportional to 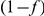.

### Extrasynaptic NMDA feedback

Evidence suggests that astrocytic released glutamate acts upon extrasynaptic NMDARs on the postsynaptic neuron to produce large SICs [23], [26]. However, the biophysical mechanism for the activation of these SICs remains unclear. To model this we propose, similar to the gatekeeper model, that glutamate (which targets and binds to extrasynaptic NMDARs) is released when Ca^2+^ levels within the astrocyte exceed 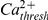. Our model for SICs is described by:
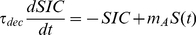
(19)where *SIC* is the NMDA slow inward current, *m_A_* controls the magnitude of *SIC*, *S* is a stimulus current used to form SIC and *τ_dec_* adjusts the decay time of SIC. S is modeled by:
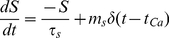
(20)where *m_s_* is the magnitude of the stimulus, τ*_S_* is the decay rate of *S* and *t_Ca_* is the time that *Ca^2+^* crosses the activation threshold from below. Research has shown that astrocytic glutamate release is correlated with Ca^2+^ oscillation peaks. Furthermore, conditions which result in a single Ca^2+^ peak followed by a sustained Ca^2+^ plateau lead to a single glutamate release [49]. To model this we assume there is only a single release of glutamate when Ca^2+^ levels exceed a threshold and no further release is possible until the threshold is crossed again from below. By adjusting the magnitude and decay time of *S* along with the decay time of *SIC* it is possible to produce a SIC with similar kinetics as those observed in CA1 neurons [21].

Research has also shown the existence of extrasynaptic α-amino-3-hydroxy-5-methyl-4-isoxazole-propionic acid receptors (AMPARs) [50], [51]. However, there is no AMPA component accompanying the activation of NMDA mediated SICs [21], suggesting that glutamate released by astrocytes targets only NMDARs. As a result, astrocytic glutamate release alone is not capable of activating NMDAR mediated SICs [23] as AMPA receptor activation is usually necessary to remove the voltage controlled magnesium NMDAR block [52]. This means that there must be a coincidental independent depolarizing stimulation to admit current through the extrasynaptic NMDARs, though the exact conditions which support such stimulation are not yet understood [23]. In our model we assume that the coincidental independent depolarizing stimulation is provided by presynaptic stimulation of the synapse via input spike trains.

To reflect these SICs in the total current supplied to the postsynaptic neuron, we modify equation (15) to:
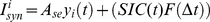
(21)where *Δt* is the time difference between the crossing of the Ca^2+^ threshold and the previous independent presynaptic stimulation. F(Δt) is given by:
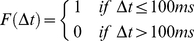
(22)From equations (21 and 22) it is clear that there will be no NMDA SIC if there is no independent presynaptic stimulus within 100 ms of the Ca^2+^ crossing. Note that 100 ms is typical of a plasticity window.

### Neuron Model

Although many neuron models exist [53] such as the Hodgkin-Huxley model [54] simplified counterparts such as the FitzHugh-Nagumo [55], [56] and Morris-Lecar models [57] are often preferred by electrophysiologists [53]. Nevertheless, these models are still computationally expensive and require a great deal of parameter tuning. Engineers and theoreticians have tended to prefer the use of the Leaky Integrate and Fire (LIF) [58], Izhikevich [59] and Spike Response [58] models as they have relatively few parameters that require tuning [53] and consequently are more suited to large network simulations [60]. For a detailed comparison of all these neural models, see [61]–[63]. The neuron model used in this work is the passive LIF model [58] described by:
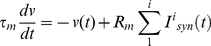
(23)where τ*_m_* is the membrane time constant, *v* is the membrane potential and *R_m_* is the membrane resistance. If *v* is greater than the firing threshold (*v_th_*) then *v* is clamped at 0 V for 2 ms; this implements the refractory period of the neuron.

### Plasticity Model

Since Donald Hebb first suggested that a neuron must fire shortly before or at the same time as a neuron to which it is connected in order to strengthen the connection between them [64], [65], there have been many mathematical models that explore synaptic plasticity (see [66] for review). However, by far the most widely accepted and phenomenologically plausible model of synaptic plasticity is Spike-Timing Dependent Plasticity (STDP) which relies on the precise time difference between pre- and postsynaptic spikes [67], [68]. If the presynaptic spike arrives before the postsynaptic spike then LTP occurs. If the presynaptic spike occurs after the postsynaptic spike then LTD occurs. Moreover, it has been shown that it is both pairs and triplets of spikes which are important; recent models show that experimental data can be well approximated when LTD is triggered by pairs of pre-post spikes while LTP is triggered via 1pre-2post triplets [69]–[72]. For present purposes it is sufficient to use a model of STDP [73] which only considers pre-post pairs for the activation of both LTP and LTD. The model is described by:
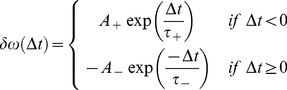
(24)where *δω*(Δ*t*) is the weight update of the synapse, Δ*t* is the time difference between pre- and postsynaptic events, *A_+_* and *A_−_* are the maximum value of weight potentiation and depression respectively and *τ_+_* and *τ_−_* control the decay rate of *δω*(Δ*t*). Equation 21 suggests that *A_se_* can be interpreted as the weight of the synapse: by varying this value it is possible to change synaptic strength. Therefore, we relate *A_se_* to *δω* and formulate an update rule as:
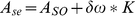
(25)where *A_SO_* is the previous synaptic weight, *δω* is the amount the weight has changed according to STDP and *K* ( = 10^−12^) is a scaling factor.

### Extension to Multiple Synapses

Recent research suggests that when employing the Li-Rinzel if IP_3_ levels become too large there is a cessation of Ca^2+^ oscillation [74]. However, there is also evidence that IP_3_ levels within the astrocyte can be degraded depending on the level of Ca^2+^ within the cytosol [39]. Results based on the Li-Rinzel model further show that under prolonged glutamate stimulation of the synapse, IP_3_ may still reach levels which cause cessation of Ca^2+^ oscillations. In contrast, it is believed that Ca^2+^ oscillations can be initiated within discrete microdomains and may be localized or propagate intracellularly by activating neighboring microdomains [17], [18], [75]–[77]. In order to model an astrocyte with multiple tripartite synapses we assume that each tripartite synapse contains a single microdomain which produces a unique Ca^2+^ oscillation based on the stimulus level present at the synapse. Although the Ca^2+^ level within the cell will differ depending on the spatial location of the stimulated microdomains and the propagation properties of the oscillations, capturing delay in the absence of supporting experimental data seems premature. Therefore, propagation delays are ignored in our model and the aggregate Ca^2+^ level remains the same everywhere throughout the cell given by:
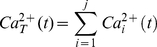
(26)where 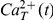 is the total level of Ca^2+^ within the cytosol at time *t* and 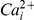 is the level of Ca^2+^ within the *i* ^th^ microdomain at time *t*.

**Figure 2:**
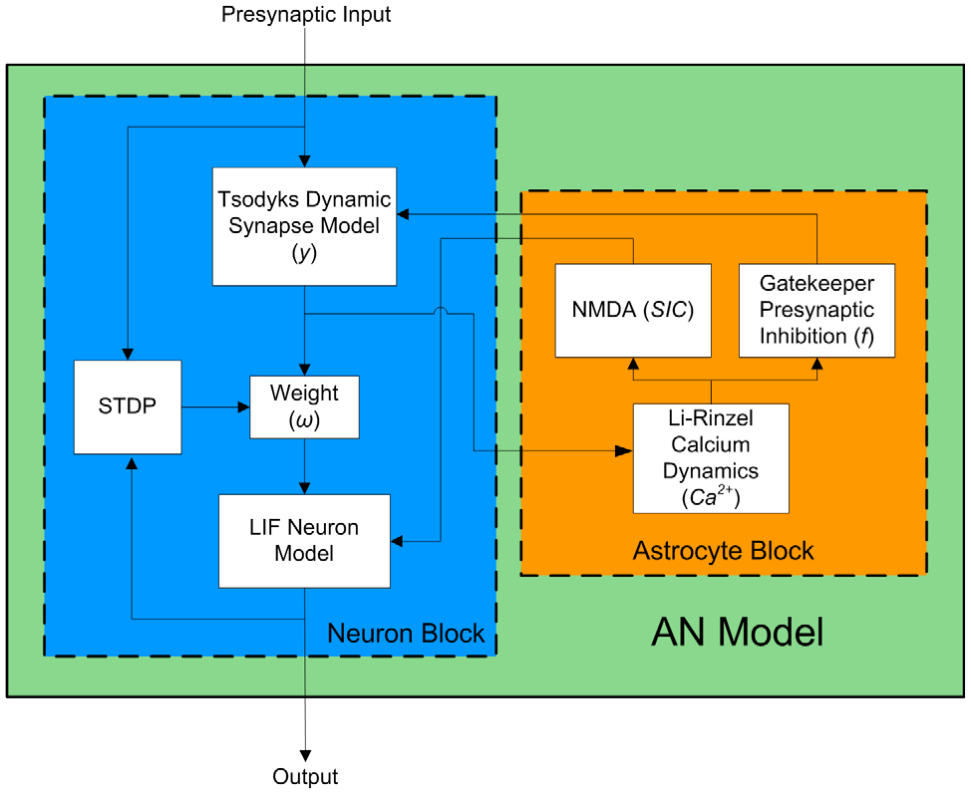
AN Model Block Diagram showing interactions between an astrocyte and neuron cell.

Given that the baseline level of IP_3_ in equation (1) is set at 0.16 µM [30] we therefore set 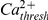 of a single synapse to just above this point at 0.18 µM. For multiple synapses we multiply this value by the number of synapses (*n*):
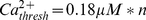
(27)
Figure 2 illustrates how the specific computational models described throughout this section are connected to form the AN model.

## Results

In all the results presented here, Matlab 2009a platform (Windows version) by Mathworks was used to realize the AN model and the Euler method of integration was used for simulation. A fixed time step of 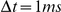 was used throughout all simulations. Simulations with a time step of 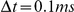 (data not shown) were also carried out and it was found that results remain unchanged.

Initial simulations provide insight into the valid range of synaptic input frequencies that cause Ca^2+^ oscillation in each of the AM, FM and AM-FM modes. This is followed by an investigation into the role of extrasynaptic NMDARs in providing a remote supervisory learning signal. Finally, we explore how the combination of pre- and post-astrocytic feedback affects coordination of neural activity. All astrocyte and synapse parameters used in these experiments can be found in Tables 1, 2 and 3 unless otherwise stated.

**Table 2:** Neuron and Synapse Parameters.

Neuron Parameter	Parameter Description	Value
*v_th_*	Firing Threshold Voltage	*9 mv*
*R_m_*	Membrane Resistance	*1.2 Gω*
*τ_mem_*	Membrane time constant	*60 ms*

Note: *v_th_* is a typical firing threshold level of a neuron and *R_m_* and *τ_mem_* have been adapted from [46] and tuned to give the desired response.

**Table 3:** Synapse Parameters.

Synapse Parameter	Parameter Description	Value
*τ_in_*	Synapse inactivation time constant	*3 ms*
*τ_rec_*	Synapse recovery time constant	*100 ms*
*τ_+_*	STDP potentiation decay time constant	*16.8 ms*
*τ_−_*	STDP depression decay time constant	*33.7 ms*
*τ_dec_*	SIC decay time constant	*37.5 ms*
*τ_s_*	SIC stimulus current decay time constant	*100 ms*
*m_A_*	Magnitude of SIC constant	*20*
*m_s_*	Magnitude of SIC stimulus constant	*20*
*u*	Utilization of synaptic efficacy	*0.1*
*A_se_*	Synaptic weight	*460–660*
*A_+_*	Maximal STDP potentiation update	*5*
*A_−_*	Maximal STDP depression update	*2.25*
*K*	Maps Ase (weight) into pA scale	*10^−12^*

Note: *τ_in_*, *τ_rec_* and *u* are taken from [30], *τ_+_* and *τ_−_* are taken from [68], *A_SE_* is set so that the neuron fires at a very low frequency for the given input and is dependent on the input stimuli to the neuron. The ratio of *A_+_* to *A_−_* are set in accordance with [68].

### Establishing the Valid Range of Input Frequencies

**Figure 3:**
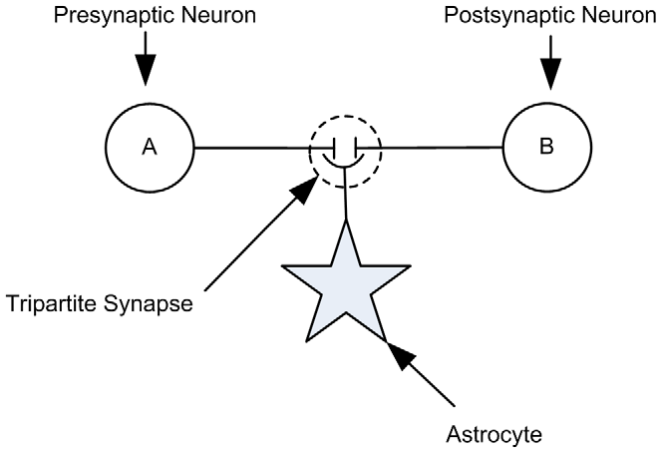
Network consists of presynaptic neuron A, postsynaptic neuron B and an interconnecting tripartite synapse. AN model is used for signaling between the tripartite synapse and astrocyte.

**Figure 4:**
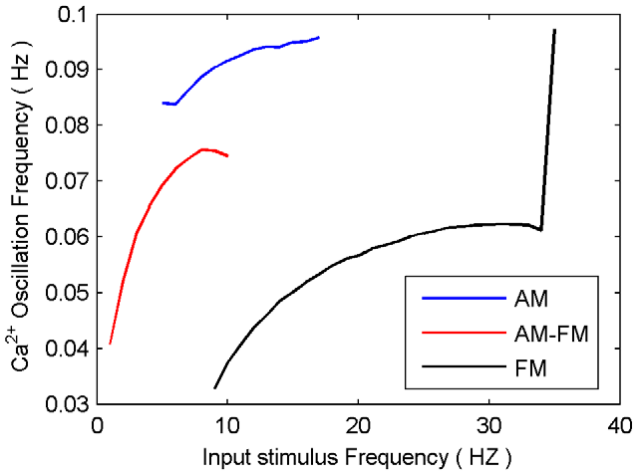
Range of input frequencies producing Ca^2+^ oscillations for each mode of operation. The valid ranges of input stimulus frequency which result in sustained Ca^2+^ oscillations are 5–17 Hz for AM, 9–35 Hz for FM and 1–10 Hz for AM-FM.

In this simulation, presynaptic neuron A stimulates a tripartite synapse with a sustained Poisson spike train for 100 s (see Figure 3). The frequency of the spike train is different for each trial and ranges from 1 Hz to 40 Hz. This is repeated for all three modes of the Li-Rinzel Ca^2+^ model i.e. AM, AM-FM and FM. The purpose of the simulation (the full AN model including the gating function *f* and NMDA SICs) is to determine the frequency ranges that result in sustained Ca^2+^ oscillation for each of the three modes. Figure 4 presents the results which show that each mode has a well-defined frequency range. In AM mode, input stimulus frequencies of between 5 Hz and 17 Hz cause sustained oscillations. In FM mode the frequency range is between 9 Hz and 35 Hz and in AM-FM mode the range is between 1 Hz and 10 Hz. In AM and FM mode, frequencies which fall below the range do not sufficiently stimulate the production of IP_3_ to enable a burst of Ca^2+^ release from the ER (see example shown in Figure S2). This is not the case in AM-FM mode due to the fact that the baseline value of IP_3_ (0.16 µM) is very close to the level at which Ca^2+^ release can occur [39]. Furthermore, frequencies which are higher than the sustained oscillation band stimulate IP_3_ production to such a degree that the negative feedback signal *f* can not prevent IP_3_ levels reaching a steady state. Consequently, oscillations fail to cross 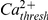 from below and above, or reach a plateau above the Ca^2+^ threshold. Examples of this can be seen in Figure S3. Unless otherwise stated, simulations presented in the remainder of this paper are from the AN model operating in the AM and AM-FM modes.

### Spatially distributed learning signals

Here we show how a learning signal, distributed spatially via an astrocyte, can promote STDP-based plasticity at remote synaptic sites.

**Figure 5:**
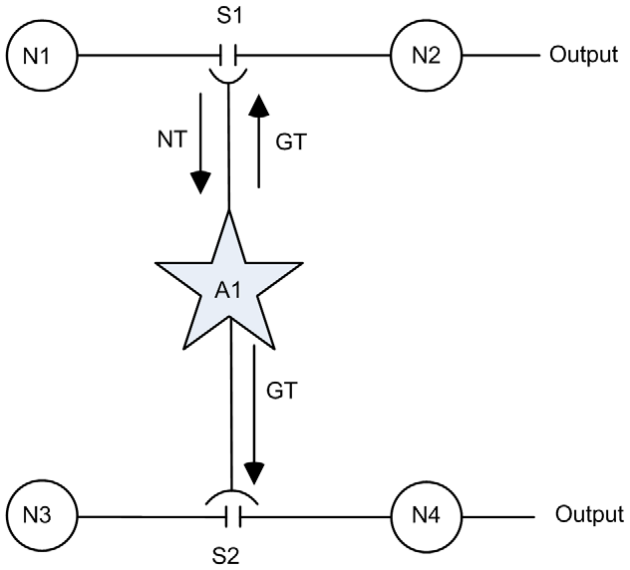
Supervised learning at S2. Network consists of pre-synaptic neurons N1 and N3, post-synaptic neurons N2 and N4 and the interconnecting astrocyte. S1 communicates bi-directionally by releasing neurotransmitter (NT) and receiving gliotransmitter (GT) while S2 only receives GT from the astrocyte.

One major requirement of STDP is that the pre- and post-synaptic neurons must fire within the plasticity window. If we consider the case where the synaptic efficacy of the synapse is insufficient to produce a postsynaptic AP then the STDP rule cannot activate learning. We propose that spatial neuron to neuron signaling using large astrocytic NMDA SICs promotes postsynaptic neuron activation. While some doubts have been expressed [78], [79] there is overwhelming evidence to suggest that astrocytes have an important role to play in synaptic plasticity [19], [20], [80]–[89]. Although further research is required [90], [91] to address their limitations, some progress can be made with existing astrocyte models. Here we use the AN model for the simple case shown in Figure 5. The network fragment consists of post-synaptic neurons N2 and N4 stimulated by independent spike trains from pre-synaptic neurons N1 and N3 via synapses S1 and S2; output spike trains from N1 and N3 last for 130 s and are Poisson-like with an average frequency of 15 Hz (this frequency was chosen because it causes a Ca^2+^ oscillation using the parameters provided in Table 1). S1 communicates bidirectionally with the astrocyte (in AM mode) as described earlier and we only consider the effects of glutamate release from the astrocyte at S2. Also weights are of sufficient magnitude to cause N2 to fire but not N4. We assumed unidirectional signalling at S2 because we are only interested in how binding of glutamate at S2 influences learning; in the network topology presented in Figure 5, the activity of S1 is sufficient to cause astrocytic release of SICs. Feedback to the astrocyte from S2 would not change the frequency of SICs, therefore we use this restricted approach to test our hypothesis.

**Figure 6:**
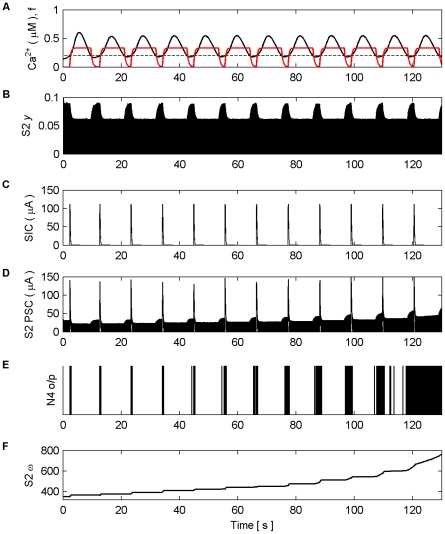
Synaptic Plasticity in the AN Model. (A) Ca^2+^ oscillation resulting from pre-synaptic stimulation of S1 by N1, including the gating function *f* (red) and Ca^2+^ threshold (dashed). (B) neurotransmitter (*y*) released by S2 as a result of pre-synaptic stimulation by N2. Note how *y* is modulated by *f* due to glutamate release by the astrocyte when Ca^2+^ levels cross the threshold from below, targeting and binding with presynaptic mGluRs. (C) NMDA-mediated SICs induced by the release of glutamate from the astrocyte when Ca^2+^ levels cross the Ca^2+^ threshold. (D) PSC (Post-Synaptic Currents) comprising EPSCs and SICs). The EPSCs in S2 are generated as a result of the neurotransmitter released by S2 scaled by the weight of the synapse (*ω*) (see F). (E) N4 output firing activity (o/p). As long as the weight of S2 remains too low, N4 is only capable of firing when the astrocyte induces an NMDA current driven by the ‘supervisory’ input of N1. However this firing promotes STDP by allowing N4 to fire and therefore S2 is potentiated (see F). From ∼45 s onward the synapse is strong enough to cause firing also as a result of the pre-synaptic activity of N3. At ∼115 s the weight quickly grows uncontrollably and the neuron begins to fire rapidly.

**Figure 7:**
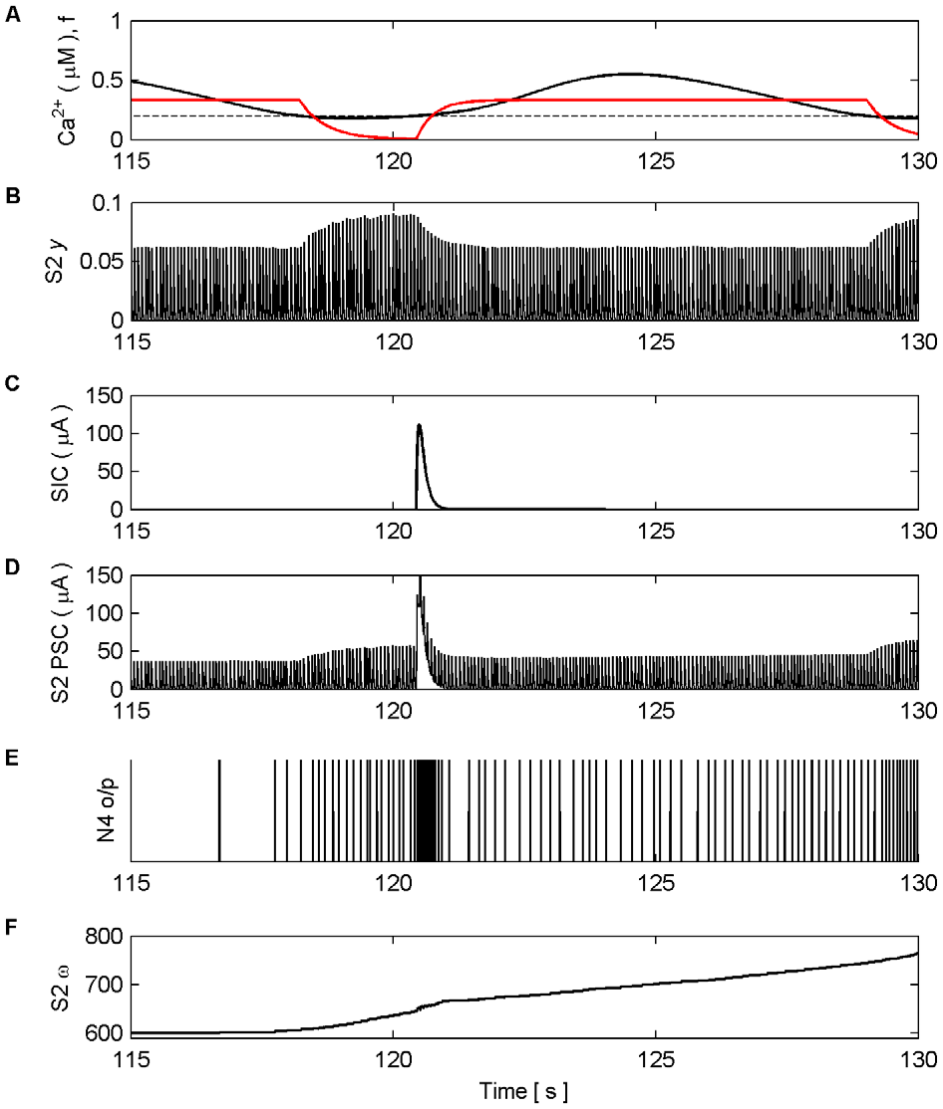
Synaptic Plasticity in the AN Model (last 15 s). (A) Ca^2+^ oscillation resulting from pre-synaptic stimulation of S1 by N1, including the gating function *f* (red) and Ca^2+^ threshold (dashed). (B) neurotransmitter (*y*) released by S2 as a result of presynaptic stimulation by N2. Again note how the amplitude of *y* is modulated by *f*. (C) SIC induced by the release of glutamate from the astrocyte when Ca^2+^ levels cross the Ca^2+^ threshold at ∼120.4 s. The kinetics of this SIC are similar to those observed in CA1 neurons [21]. (D) PSCs at S2, comprising EPSCs elicited by the neurotransmitter released by N2 and the SIC. (E) N4 output firing activity. (F) Synaptic weight (*ω*). At ∼115 s the synapse is strong enough to cause firing as a result of the presynaptic activity of N3 without the aid of NMDA induced SICs.

Figure 6A shows the Ca^2+^ oscillation within the astrocyte and the gating function *f*. When the Ca^2+^ oscillation passes 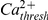 (dashed line) from below glutamate, which targets presynaptic mGluRs and extrasynaptic NMDARs, is released from the astrocyte to both S1 and S2. This induces large SICs in both synapses at frequencies equal to that of the Ca^2+^ oscillation (Figure 6C). Figure 6D shows the total PSC, which consists of the SIC and the presynaptically induced EPSC that result from the level of neurotransmitter (*y*) released by the axon (Figure 6B) scaled by the weight of the synapse (ω) (Figure 6F). It is clear that each time the astrocyte releases glutamate there is a sharp rise in the PSC (Figure 6D) due to the large NMDA induced currents (SIC). It is also clear that the release of glutamate which targets mGluRs modulates the amount of neurotransmitter released from the axonal bouton; this modulation mirrors the kinetics of *f*. The firing rate of N4 is synchronized to SIC below 115 s but thereafter the firing rate of N4 increases due to presynaptic activity alone, as shown also in Figure 6E. Figure 6F shows the synaptic weight evolution of S2. It can be seen that the weight potentiates on S2 as a result of the STDP rule, and that after ∼115 s the synapse has potentiated sufficiently to initiate a causal relationship between N3 and N4; increasing weight correlates with the firing rate of N4. This can be seen more clearly in Figure 7 which depicts the last 15 s of the model simulation.

**Figure 8:**
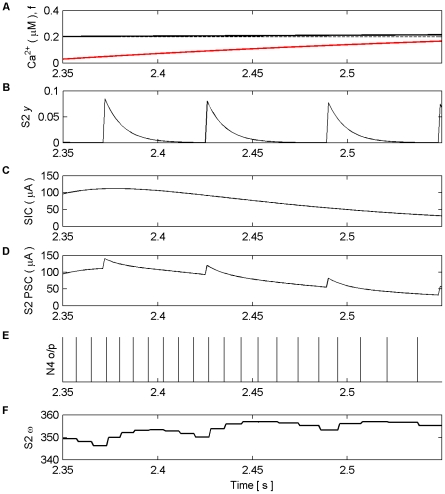
Synaptic Plasticity in the AN Model (2.35 s–2.55 s). (A) Ca^2+^ oscillation resulting from pre-synaptic stimulation of S1 by N1, including the gating function *f* (red) and Ca^2+^ threshold (dashed). (B) neurotransmitter (*y*) released by S2 as a result of presynaptic stimulation by N2. (C) SIC induced by the release of glutamate from the astrocyte when Ca^2+^ levels cross the Ca^2+^ threshold (crossing point not shown). (D) PSCs at S2, comprising EPSCs elicited by the neurotransmitter released by N2 and the SIC. (E) N4 output firing activity. (F) Synaptic weight (*ω*), Note that the weight either potentiates or depresses based on the temporal order of the pre and post neural activity.

On initial inspection of this result it appears that only potentiation occurs as a result of the learning rules. However, closer inspection of Figure 6 shows that depression also occurs as a result of the temporal order of input and output spikes. This can be seen in Figure 8 which shows results of this simulation from 2.35 s to 2.55 s. Furthermore, we envisage that in the case of multiple synapses the temporal order of spikes would dictate which synapses depress and which potentiates.

### Dynamic Coordination

**Figure 9:**
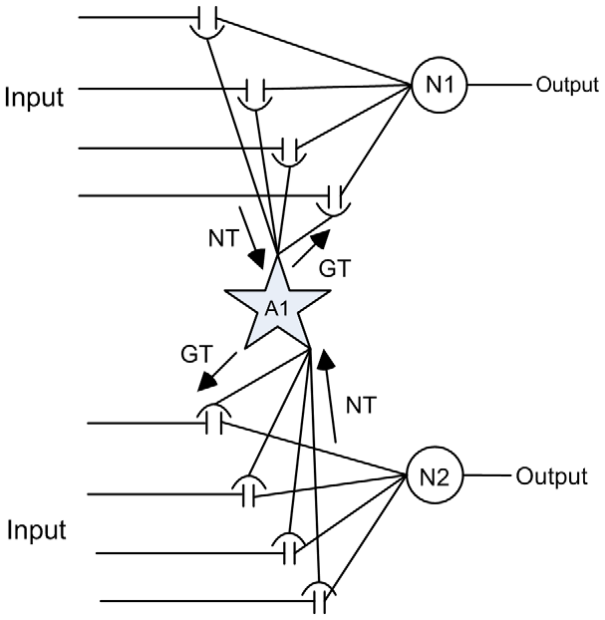
Dynamic coordination in the AN model. All synapses connected to the astrocyte can communicate via bidirectional signaling of neurotransmitter (NT) and gliotransmitter (GT).

Neural oscillations across a broad range of frequency bands are ubiquitous throughout the nervous system and give rise to a wide variety of dynamic coordination effects including synchrony, learning, precisely timed phase-shifts among oscillating neural ensembles, concatenation of different rhythms, and so forth [92]–[96]. Given that glutamate released by astrocytes can activate synchronized SICs in neighboring synapses thereby acting as a bridging mechanism between circuits which are not directly coupled [97], [98], we now investigate dynamic coordination using the network in Figure 9 where each of the neighboring neurons N1 and N2 has four tripartite synapses. In these simulations, synapses associated with both N1 and N2 communicate in a bidirectional manner. Please note that in these simulations we do not consider the effects of synaptic plasticity and as such do not apply the plasticity rules as given by equation 24.

**Figure 10:**
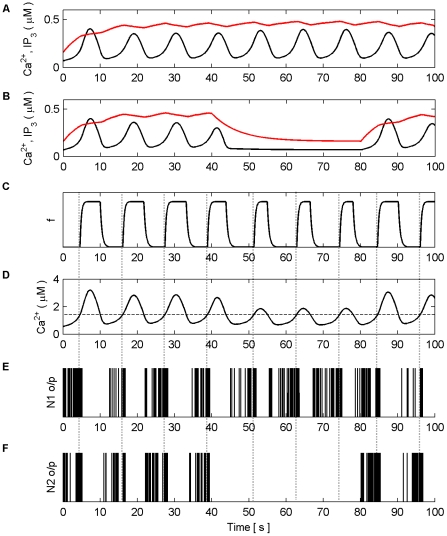
Ca^2+^ oscillations and resulting coordination. (A) Ca^2+^ oscillations (black line) and IP_3_ levels (red line) at a synapse from N1 stimulated by 7 Hz Poisson spike train for 100 s. (B) Ca^2+^ oscillations at a synapse from N2 which is only stimulated from 0–40 s and 80–100 s with a 7 Hz Poisson spike train. (C) Activity of the gating function *f* which is activated when the total level of Ca^2+^ (D) within the astrocyte passes the threshold (D-black dashed line). (E and F) The output firing activity of neurons N1 and N2. Note that when the total Ca^2+^ oscillation crosses the threshold from below both neurons fire with a significantly higher frequency of activity; a result of the global release of glutamate and NMDA SIC activation. These are the only times that the neurons are highly coordinated. Furthermore it can be seen that there are extended periods of silence from both neurons after firing in bursts. This is a result of the negative feedback from *f* which depresses the release of neurotransmitter from the synapses and remains active until the Ca^2+^ oscillation crosses the threshold from above.

Firstly, we explore how the model produces coordination when all the synapses are subjected to the same stimulus within the valid frequency range (see Figure 4). The synapses connected to N1 were stimulated for the duration of the experiment (100 s), while the synapses associated with N2 were stimulated from 0 to 40 s and 80 to 100 s. The individual synaptic weights of both N1 and N2 were set at a level which resulted in postsynaptic firing of the connected neuron when all synapses were stimulated with a series of spikes (spike trains). Figure 10 (A–B) shows an example of the resulting Ca^2+^ oscillations and IP_3_ levels produced by Poisson spike trains with an average frequency of 7 Hz stimulating the synapses of N1 (A) and N2 (B) while in AM mode. Given that there is a microdomain associated with each synapse, the response of which will be very similar to the same Poisson stimulus, Figure 10 (A) shows the response of a single microdomain to a stimulus from a synapse associated with N1 while (B) is the response of a microdomain to a stimulus from a synapse associated with N2. (C) is the *f* function. Figure 10 (D) shows the aggregate Ca^2+^ level within the astrocyte while (E) and (F) describe the output firing activity of N1 and N2 as a result of the AN model. Observe that while the background firing activity of N1 and N2 is not coordinated, both N1 and N2 burst at the same time provided presynaptic inputs are present at N2 (Note: we define a burst as a period of neural activity with a significantly higher firing frequency [99]). This coordinated activity happens exactly at the same time because we have ignored signal propagation delays.

**Figure 11:**
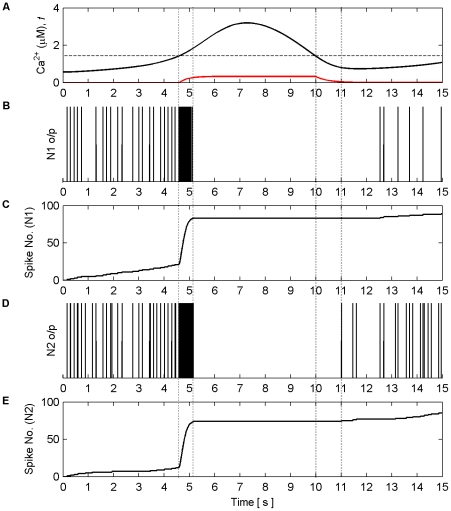
Coordinated firing activity of N1 and N2 for the first 15 s of AN model simulation. (A) The aggregate level of Ca^2+^ (black line) and gating function *f* (red line). (B) and (D) firing activity of N1 and N2. (C) and (E) firing activity of N1 and N2 plotted as number of output spikes against time. When the level of Ca^2+^ crosses the threshold (1^st^ vertical dashed line) SIC stimulates all synapses causing each neuron to burst for approximately 600 ms (2^nd^ vertical dashed line). During this time the gating function *f* depresses neurotransmitter release from all synapses, during which the neurons are held silent as a result of decreased neurotransmitter release, until the Ca^2+^ level crosses the threshold from above (3^rd^ vertical dashed line) after which it takes approximately 1 s for the gating function to fully stop synaptic depression (4^th^ vertical dashed line). When this period ends the neuron can again fire as the synapses are releasing transmitter fully.

It is interesting to note that when the Ca^2+^ threshold (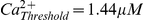) is crossed from below there is a burst of firing activity from both neurons. This is a result of the astrocyte coordinated SIC signals at all synapses. Note the silent periods after bursting which are a result of the negative feedback provided by mGlu modulation of pre-synaptic transmission controlled by *f* (Figure 10C). It should also be noted that during silent periods N2 does not respond to the SICs because post-synaptic excitation due to pre-synaptic firing is also required for N2 to fire. Also, as a result of there being no stimulation of N2 synapses between 40 s and 80 s, IP_3_ is no longer created at these synapses and is allowed to degrade: Ca^2+^ oscillations cease during this period. This is evident from Figure 10D where it can be seen that the aggregate level of Ca^2+^ falls significantly between 40 and 80 s. However it still remains high enough to cause passing of the threshold and therefore the activation of SICs and the gating function *f* continues during this period. This can be seen much more clearly in Figure 11 where (A) represents the aggregate level of Ca^2+^ within the cell (black line) and the gating activity of *f* (red line). Figure 11 (B) and (C) show the number of spikes output from neurons N1 and N2 respectively against time.

This type of simulation was repeated for all frequencies within the valid frequency range (see Figure 4) of both AM and AM-FM modes: in every case the neural activity was produced in the same manner. The only significant difference found in AM mode was that the higher the stimulus frequency the earlier the onset of coordination. This was not the case in the AM-FM mode, however, the time between coordinated bursts was found to vary as a result of the variations of frequency of oscillation for each of the different input stimuli.

**Figure 12:**
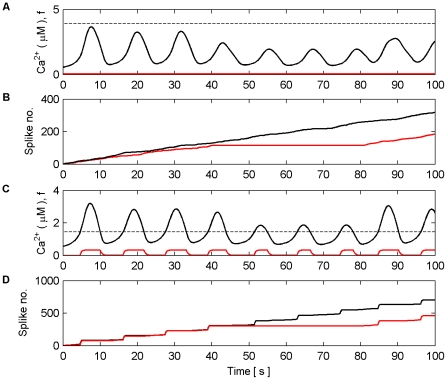
Example of total Ca^2+^ oscillations and neural coordination with different Ca^2+^ threshold levels. (A) Ca^2+^ threshold (dashed line) set at 3.94 µM. (B) Neural activity of N1 (black) and N2 (red) plotted as number of spikes against time with the Ca^2+^ threshold set at 3.94 µM. (C) Ca^2+^ threshold set at 1.44 µM. (D) Same as (B) except that the Ca^2+^ threshold is 1.44 µM. The input stimuli to the synapses for both simulations are set at 7 Hz. When the threshold is too high (A) there is no crossing of the threshold by the total level of Ca^2+^. Since there is no astrocytic global release of glutamate, *f* is not activated and there is no coordinating bursting of the neurons (B). In contrast, where the threshold is crossed (C) and (D), *f* is activated and NMDA SICs are induced (not shown) thus producing coordinated activity of N1 and N2. Note that in both cases there is a reduction in total Ca^2+^: N2 produces no firing activity because the N2 synapse receives no stimulus between 40 s and 80 s.

Since the Ca^2+^ threshold within our model can be treated as an open parameter we investigated its effect on coordination. The same simulations as previously presented were again repeated with the threshold varying from 1.44 µM to 3.94 µM in steps of 0.5 µM per trial. The Ca^2+^ threshold was found to have a significant impact on the coordination of the neurons. Firstly, if the threshold was beyond the level reached by the total Ca^2+^ oscillation, no coordination in the form of synchronized bursts from N1 and N2 occurred. Figure 12 compares the total Ca^2+^ oscillation and neural coordination when the threshold is set at 3.94 µM and 1.44 µM with all synapses stimulated with a 7 Hz Poisson spike train.

**Figure 13:**
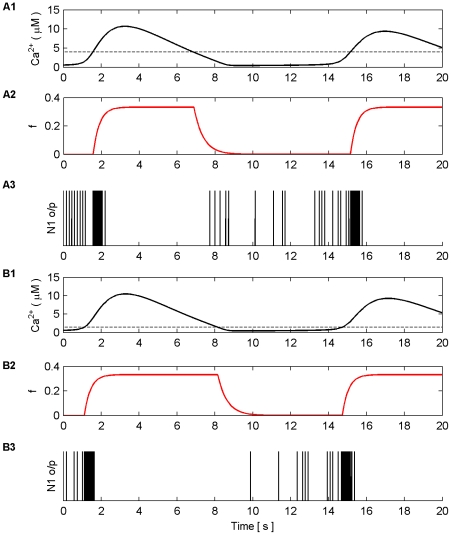
Threshold variations vs the onset of coordination. (A1) Total Ca^2+^ level and Ca^2+^ threshold (dashed line, 3.94 µM). (A2) *f* function. (A3) Spiking activity (o/p) of N1. (B1–B3) same as (A1–A3) with Ca^2+^ threshold set at 1.44 µM. Notice how the onset of bursting and the *f* function is delayed when the threshold is set at 3.94 µM since more time is required for the total Ca^2+^ level to cross the threshold. Furthermore, *f* is activated for a shorter period thereby reducing the duration of the silent period between bursts. Note that N2 bursts at the same time as N1 (data not shown).

Secondly, as the Ca^2+^ threshold was raised it was found that the timing of the onset of the first burst in both the AM and AM-FM modes was changed: the higher threshold delayed the onset. In both cases the initial burst was followed by bursting that was synchronized to the total Ca^2+^ oscillation. Furthermore, in both cases the activity of N1 and N2 changed between the periods of bursting activity: as the threshold was increased the *f* function was activated for much shorter periods. Therefore, the duration of the silent periods of neural activity was reduced as a result of shorter periods of depression by *f*. Figure 13 shows an example of this phenomenon where the model is in AM-FM mode and all synapses are stimulated with a 7 Hz Poisson spike train.

**Figure 14:**
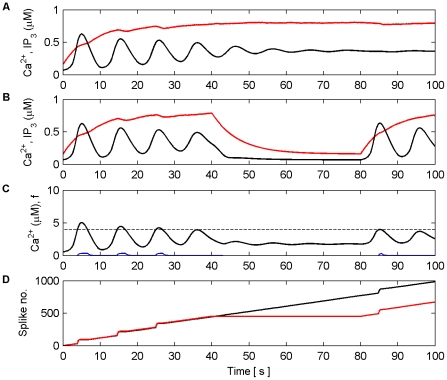
Loss of Ca^2+^ oscillation in the synaptic microdomain. (A) Ca^2+^ oscillation (black) and IP_3_ levels (red) within a synaptic microdomain of N1. (B) Ca^2+^ oscillation and IP_3_ levels within a synaptic microdomain of N2. (C) Total Ca^2+^ level within the astrocyte as a result of all synaptic microdomain oscillations and the *f* function (blue).(D) Neural output of N1 (black) and N2 (red) as spike count as a function of time. Since the threshold is close to the peak of the total Ca^2+^ oscillation *f* is only active for a short period. Therefore the reduction of IP_3_ due to synaptic neurotransmitter release depression cannot prevent IP_3_ levels reaching a point at which the sustained Ca^2+^ oscillations cease at synapses associated with N1. This is not the case at synapses associated with N2 as there is no synaptic input stimulation between 40 s and 80 s and IP_3_ levels degrade naturally. Furthermore, the coordinated activity also changes: between 40 s and 80 s there is no bursting activity in either neuron since the total Ca^2+^ level is insufficient to cross the threshold.

Reduction in synaptic depression was also found to change the valid frequency range. As the threshold was increased it was found that when frequencies at the upper limit of the valid input frequency range were used to stimulate the synapse, the reduction in synaptic depression could not prevent the synapses associated with N1 from producing saturated IP_3_ levels and the individual Ca^2+^ oscillations of these synapses ceased. Figure 14 depicts the case of 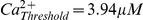 with all synapses stimulated at 14 Hz.

**Figure 15:**
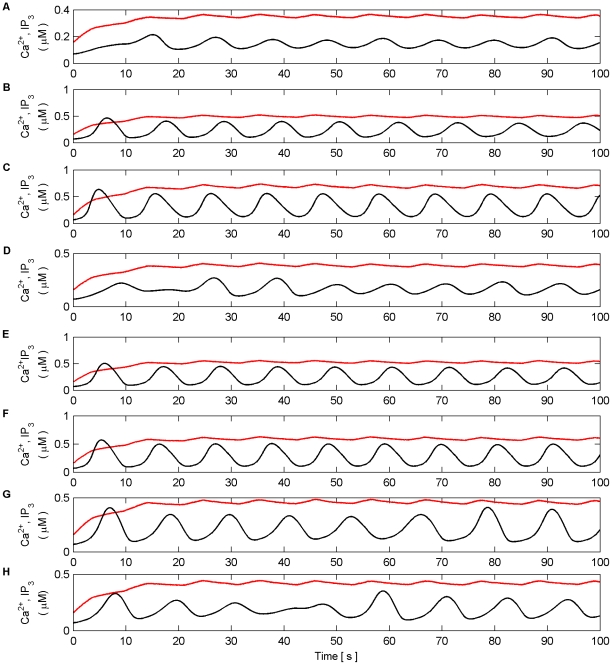
Individual Ca^2+^ oscillations and IP_3_ levels in AM mode. (A–H) Individual Ca^2+^ oscillations (black), IP_3_ transients (red) in each of the 8 microdomains associated with the synapses of N1 (A–D) and N2 (E–H). The input stimulus to each microdomain is a Poisson spike train with an average frequency of (A) 5 Hz, (B) 9 Hz, (C) 15 Hz, (D) 6 Hz, (E) 10 Hz, (F) 12 Hz, (G) 8 HZ, (H) 7 Hz. Note how the phase of the individual oscillations change, especially in (A), (D) and (H). Note also that the oscillation of IP_3_ occurs at the same time for all microdomains. This is a result of the *f* function triggered by total Ca^2+^ which regulates the release of neurotransmitter at all synapses. It is the global oscillation of IP_3_ that causes the phase shift of the individual Ca^2+^ oscillations.

**Figure 16:**
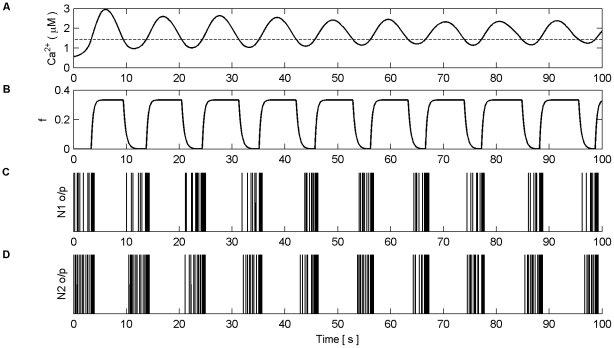
Total Ca^2+^ oscillation and neural firing activity in AM mode. (A) Total Ca^2+^ oscillation and Ca^2+^ threshold (dashed line). (B) *f* function. (C) Neural firing activity of N1. (D) Neural firing activity of N2. Note how total Ca^2+^ is much more sinusoidal than in previous experiments and that coordinated bursting of N1 and N2 occurs each time Ca^2+^ crosses the threshold from below.

Next we investigated dynamic coordination when Poisson spike trains with different average frequencies were applied to each synapse of N1 and N2 in both modes. Figures 15 and 16 present the results of this simulation in AM mode. Frequencies were chosen arbitrarily to be 5 Hz, 9 Hz, 15 Hz, and 6 Hz for synapses associated with N1 and 10 Hz, 12 Hz, 8 Hz and 7 Hz for synapses associated with N2. All synapses were stimulated for the duration of the simulation (100 s). Figure 15 (A–H) shows the individual Ca^2+^ oscillation and IP_3_ transients of each synaptic microdomain while Figure 16 (A) shows the total Ca^2+^ oscillation, (B) the *f* function and (C–D) the output firing activity of N1 and N2. From these results we note that the phases of the individual Ca^2+^ oscillations are changed. This is especially evident from Figure 15 (H). Note also that the total Ca^2+^ is much more sinusoidal than in previous experiments yet coordination is still maintained.

**Figure 17:**
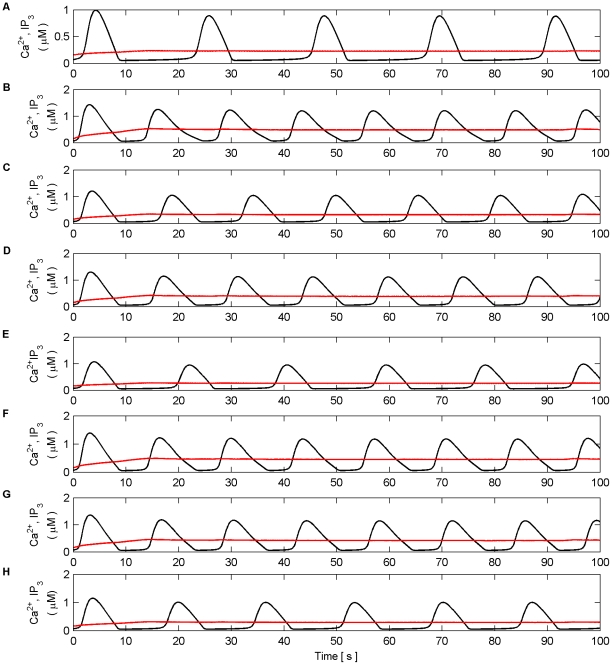
Individual Ca^2+^ oscillations and IP_3_ levels in AM-FM mode. (A–H) Individual Ca^2+^ oscillations (black), IP_3_ transients (red) in each of the 8 microdomains associated with the synapses of N1 (A–D) and N2 (E–H). The input stimulus to each microdomain is a Poisson spike train with an average frequency of (A) 2 Hz, (B) 10 Hz, (C) 5 Hz, (D) 7 Hz, (E) 3 Hz, (F) 9 Hz, (G) 8 HZ, (H) 4 Hz. Note that there is no noticeable phase locking of the individual Ca^2+^ oscillations.

**Figure 18:**
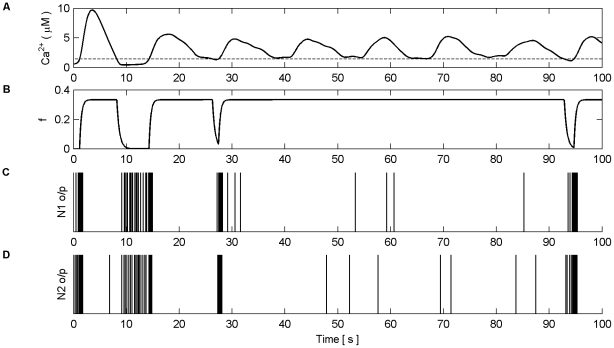
Total Ca^2+^ oscillation and neural firing activity in AM-FM mode. (A) Total Ca^2+^ oscillation and Ca^2+^ threshold (dashed line). (B) *f* function. (C) Neural firing activity of N1. (D) Neural firing activity of N2. Note how total Ca^2+^ is much more erratic. As a result the coordinated bursting of N1 and N2 is less frequent since the total level of Ca^2+^ crosses the threshold from below less often. Furthermore, it can be seen that *f* is activated for a much greater period of time (e.g. between 28 s and 94 s) since the total level of Ca^2+^ does not cross the threshold from above.

Figures 17 and 18 present model simulations in the AM-FM mode. Frequencies were again chosen arbitrarily to be 2 Hz, 10 Hz, 5 Hz, and 7 Hz for synapses associated with N1 and 3 Hz, 9 Hz, 8 Hz and 4 Hz for synapses associated with N2. Once more the inputs to all synapses were maintained for the duration of the experiment (100 s). Figure 17 (A–H) shows the individual Ca^2+^ oscillation and IP_3_ transients of each synaptic microdomain while Figure 18 (A) shows the total Ca^2+^ oscillation, (B) the *f* function and (C–D) the output firing activity of N1 and N2. From these results we note that there is no noticeable phase locking of the individual Ca^2+^ oscillations: the coordination of N1 and N2 is not maintained at regular periods as in the AM mode due to the irregular total Ca^2+^ level. The results of these simulations suggest that the negative feedback and IP_3_ regulation provided by the *f* function help maintain the coordination of N1 and N2. Phase locking of the individual Ca^2+^ oscillations appears to occur and the total Ca^2+^ oscillation resembles a sinusoid. Further simulations in which the *f* function was removed revealed in all cases that coordination was lost and the total Ca^2+^ oscillation became much less sinusoidal.

**Figure 19:**
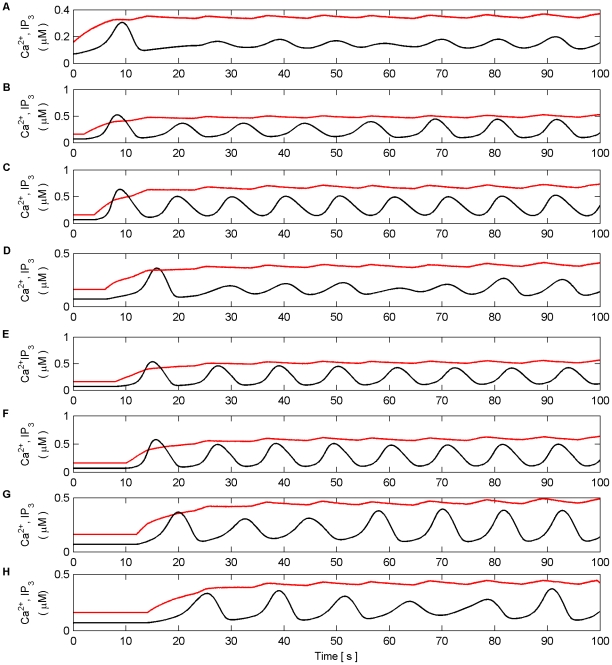
Individual Ca^2+^ oscillations and IP_3_ levels in AM mode. (A–H) Individual Ca^2+^ oscillations (black), IP_3_ transients (red) in each of the 8 microdomains associated with the synapses of N1 (A–D) and N2 (E–H). The input stimulus to each microdomain is a Poisson spike train with an average frequency of (A) 5 Hz (0 s–100 s), (B) 9 Hz (2 s–100 s), (C) 15 Hz (4 s–100 s), (D) 6 Hz (6 s–100 s), (E) 10 Hz (8 s–100 s), (F) 12 Hz (10 s–100 s), (G) 8 HZ (12 s–100 s), (H) 7 Hz (14 s–100 s). Note how the phase of the individual oscillations phase lock each microdomain Ca^2+^ oscillation.

**Figure 20:**
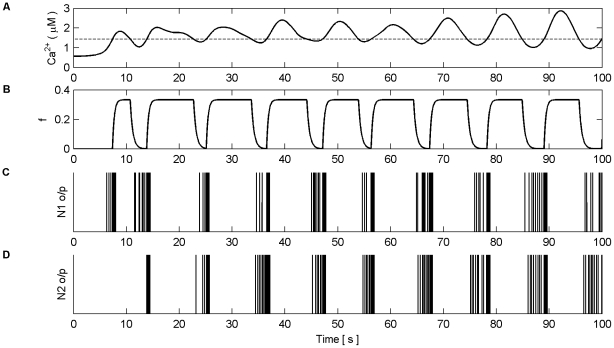
Total Ca^2+^ oscillation and neural firing activity in the AM mode. (A) Total Ca^2+^ oscillation and Ca^2+^ threshold (dashed line). (B) *f* function. (C) Neural firing activity of N1. (D) Neural firing activity of N2. Note how total Ca^2+^ is much more erratic up until ∼40 s. As a result, the coordinated bursting of N1 and N2 during this time does not have a constant period. After ∼40 s phase locking of the individual microdomains is achieved and the coordinated activity of N1 and N2 is more constant. Furthermore, the total Ca2+ oscillation once again becomes much more regular.

Given the foregoing results suggest phase locking of individual Ca^2+^ oscillations, the previous two simulations were repeated with all synaptic stimuli in succession with a delay of 2 s between each stimulus. Despite the fact that the phase of each individual Ca^2+^ oscillation is now different, in AM mode it was found that the individual oscillations are successfully phase locked (Figure 19) and that coordination of N1 and N2 is maintained (Figure 20). Furthermore the pattern of coordination is also changed. From Figure 20 it can be seen that the periods between bursting of N1 and N2 are not constant.

**Figure 21:**
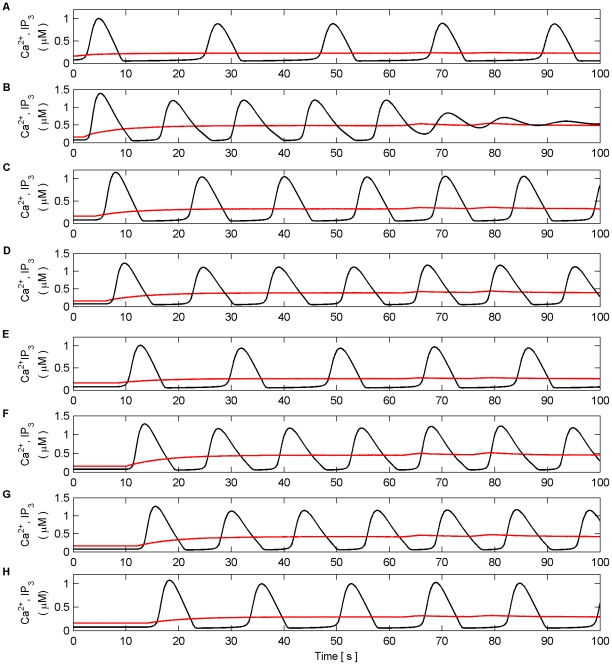
Individual Ca^2+^ oscillations and IP_3_ levels in the AM-FM mode. (A–H) Individual Ca^2+^ oscillations (black), IP_3_ transients (red) in each of the 8 microdomains associated with the synapses of N1 (A–D) and N2 (E–H). The input stimulus to each microdomain is a Poisson spike train with an average frequency of (A) 2 Hz, (B) 10 Hz, (C) 5 Hz, (D) 7 Hz, (E) 3 Hz, (F) 9 Hz, (G) 8 HZ, (H) 4 Hz. Note there is no phase locking of the individual microdomain oscillations.

**Figure 22:**
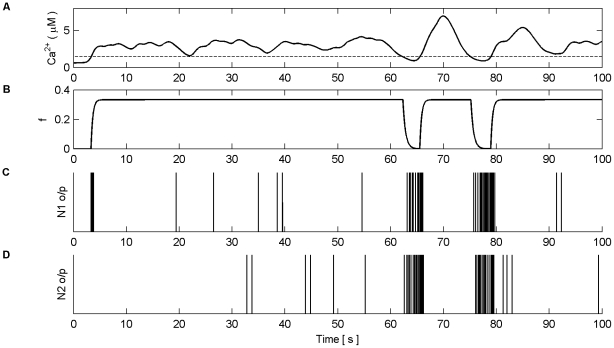
Total Ca^2+^ oscillation and neural firing activity in the AM-FM mode. (A) Total Ca^2+^ oscillation and Ca^2+^ threshold (dashed line). (B) *f* function. (C) Neural firing activity of N1. (D) Neural firing activity of N2. Note how total Ca^2+^ is much more erratic and infrequently crosses the threshold from above and below. Although the *f* function is activated for much longer periods, negative feedback is still unable to cause phase locking. As a result the coordinated bursting activity of N1 and N2 is significantly reduced.

In the AM-FM mode no phase locking of individual waves was accomplished (Figure 21). The total Ca^2+^ oscillation is much less sinusoidal and the pattern of oscillation is different. Moreover, coordination is sporadic since the total Ca^2+^oscillation infrequently crosses the threshold from above and below (Figure 22).

Finally we investigated the extent to which phase locking can occur between different Ca^2+^ oscillations in both modes and its concomitant effects on coordination. For this investigation we reduced the number of synapses on each output Neuron (N1 and N2) to one synapse. We then investigated each combination of input stimulus frequency (in steps of 1 Hz) and different phases by repeating each experiment with a different starting time for the synapse of N2 (start time = 0 s–10 s, in steps of 2 s) for the valid input frequency range of each mode. In the AM mode it was found that phase locking of the individual waves was achieved for the entire valid input frequency range as long as the phases were not 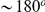 out of phase. In AM-FM mode it was found that phase locking was only achieved when the individual Ca^2+^ oscillations were between 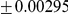 Hz and no more than 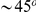 out of phase. In both modes coordination was still possible even when phase locking could not be achieved as long as the total Ca^2+^ oscillation still crossed the threshold from above and below. However, coordination of the N1 and N2 was often infrequent and not maintained.

## Discussion

The AN model presented in this paper captures the bidirectional coupling between astrocytes and neurons and in so doing demonstrates that positive and negative feedback to extrasynaptic NMDARs and presynaptic mGluRs significantly contributes to the regulatory capability of astrocytes.

### Astrocytic ‘supervisory’ learning signal

Experimental evidence indicates that astrocytes have a role to play in LTP/LTD. Deficiency of GFAP (glial fibrillary protein), which is predominantly expressed by astrocytes in the CNS, has been found to enhance LTP and impair LTD [19], [20]. For many years STDP has been accepted as one of the most popular and biological plausible mechanisms of synaptic plasticity. However, it is dependent on presynaptic input and postsynaptic output activity to induce plasticity changes. Our results show that it is possible to initiate STDP learning when the weight of the synapse is too weak to cause post-synaptic neural firing. When astrocytic Ca^2+^ levels rise above a threshold due to stimulation from a neuron or neurons, glutamate is released which in turn activates large SICs which can induce postsynaptic firing in other neurons, thereby causing STDP related weight potentiation/depression. This learning relies on a presynaptic stimulus and astrocytic induced SICs. However, after the synaptic weight is strengthened significantly, presynaptic stimulation alone is sufficient to cause firing of the presynaptic neuron and the synaptic weight grows uncontrollably. Such instability is in agreement with other research [73], [100], [101] and a mechanism for weight capping has been proposed based on the Bienenstock Cooper Munro (BCM) rule [102]. Specifically, it has been shown that merging BCM with STDP can create stability in plasticity. However, much research is still required to clearly establish the biophysical underpinnings of these rules [103], [104]. Despite this shortcoming the present modeling research points to a new mechanism of plasticity based on the interactions between astrocytes and neurons.

When the postsynaptic neuron is sufficiently depolarized voltage gated-channels allow the influx of Ca^2+^ into the dendrite causing endocannabinoids to be synthesized and subsequently released from the dendrite. However, the exact release machinery related to this process is not fully understood [105]. Endocannabinoids are a type of retrograde messenger which travel back from the postsynaptic membrane to the pre-synaptic terminal. The release of 2-arachidonyl glycerol (2-AG), a type of endocannabinoid, is known to feed back to the pre-synaptic terminal directly and indirectly via an astrocyte. The direct route results in a decrease in transmission probability (PR) and is termed Depolarization-induced Suppression of Excitation (DSE) [105], while the indirect route results in the astrocytic release of glutamate which binds to pre-synaptic group I mGluRs and gives rise to an increase of synaptic transmission probability termed e-SP [106]. Note that the indirect signaling pathway is far reaching and can affect distant synapses [106]. It is therefore plausible that these opposing mechanisms could modulate the probability of release, and therefore the firing activity of pre and postsynaptic neurons, thereby affecting a Hebbian-like learning process. Our AN model may be extended to more complex networks where the training signal (i.e. the astrocyte-derived SIC) is able to fuse sensory inputs across remote neuron clusters. In doing so we would effectively be investigating how structural/synaptic plasticity in large networks of neurons may be regulated by astrocytes. However, for such experiments to proceed and succeed, more information is needed regarding signal transmission across the gap junctions associated with astrocytes.

### Dynamic coordination

The AN model also shows that astrocytes have a key role in the dynamic synchronization of neurons. The SICs and neurotransmitter modulation induced by astrocytic release of glutamate causes coordinated neural activity in remote neurons. Our results suggest that the frequency of input stimuli, the phase relation of the astrocytic Ca^2+^ oscillations and the Ca^2+^ threshold responsible for the release of gliotransmitter directly impact on the pattern of synchronization. Extrasynaptic NMDAR activation was needed to provide coordinated periods of bursting activity. We also found that negative feedback of presynaptic mGluR activation was important for maintaining neural coordination via phase locking of the Ca^2+^ oscillations. In exploring the effects of out of phase oscillations we expected the total Ca^2+^ oscillation to be a complex wave comprising the sum of the individual waves. These should be out of phase due to the different initiation times of the input spike trains. However, our results suggest that over a period of time and under certain conditions Ca^2+^ oscillations may become phase locked due to the negative feedback provided by *f*. The latter arises from modulation of neurotransmitter in the cleft of all synapses associated with the astrocyte and consequently the IP_3_ level in the astrocyte. This process leads to gradual alignment of Ca^2+^ oscillations in each microdomain that, when successful, provides a simple sinusoidal like total Ca^2+^ oscillation. Moreover, it has recently been suggested that oscillations within microdomains of astrocytic distal processes differ from oscillations at the soma [107]. In our model we linearly sum the individual microdomain oscillations to create a total Ca^2+^ oscillation which may be considered to occur at the soma. Despite this simplistic assumption our results show that when phase locking cannot be achieved, oscillations within the distal processes and the soma are indeed different.

Our results also suggest that even though phase locking can occur it is not vital for coordination. In AM mode, when both waves were approximately in antiphase, phase locking could not be achieved, the total Ca^2+^ oscillation becoming much flatter and less sinusoidal. However, when both input stimulus frequencies were below 12 Hz and again in antiphase, coordination was still possible. The reason is that lower frequencies produce lower amplitudes: as a result, it was still possible for the fluctuation of Ca^2+^ to cross the threshold and release glutamate, thus producing global SICs responsible for coordination. On the other hand, when both frequencies were above 12 Hz and in antiphase, coordination was no longer possible as the fluctuations of the total Ca^2+^ were not sufficient to continually cross the Ca^2+^ threshold. Consequently, no further global release of glutamate from the astrocyte was possible and the SICs died off. In AM-FM mode phase locking occurred much less frequently and was only achieved when the individual Ca^2+^ oscillations were between 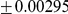 Hz and no more than 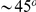 out of phase. Again, coordination was still possible as long as total Ca^2+^ oscillation crossed the threshold from above and below.

Although propagation delays were not included in the present version of the AN model it would be interesting to investigate intercellular delays and the propagation time for waves across astrocyte networks. For example, it is likely that dynamic coordination across neuron clusters, mediated via astrocyte networks, is important for the brain rhythms underlying cognitive function. Dynamically altering the cluster size and with that their spatial location may mean that the Ca^2+^ signal pathways are dynamically changing causing ongoing phase shifting between bursting activity across the clusters. If we assume that the “envelope” of a rhythm correlates with the times of maximum bursting then the frequency of the brain oscillation depends on cluster locations and consequently on delays. Moreover, the phase locking characteristics of our model may also provide a mechanism for dynamically changing the coordination between neuron ensembles. Since phase locking was found to occur between microdomain oscillations with approximately the same frequency of oscillation, it is not inconceivable that stimuli of similar frequency may cause a unique pattern of oscillation which changes as the stimuli frequencies change.

Our model shows that the Ca^2+^ threshold responsible for releasing glutamate from the astrocyte has an effect on the valid input frequency range that causes Ca^2+^ oscillations in each microdomain. Furthermore, this threshold may also have an impact on the pattern of coordination between neurons. When phase locking occurs the total Ca^2+^ oscillation is sinusoidal like and therefore the bursting activity is regular and periodic. In both modes many of the higher input frequencies caused the total Ca^2+^ oscillation to cease crossing the threshold. This condition may be avoided, however, if the threshold is raised. More biological experimentation is required to establish the key parameters governing the threshold level.

Ca^2+^ oscillations are related to the frequency of the input stimuli and dependent on IP_3_ and its rate of change (*r_ip3_*) which, in our model, is not constrained and therefore can be treated as an open parameter. Therefore, changing the value of *r_ip3_* would make the Ca^2+^ oscillations sensitive to a band of different input frequencies thereby providing a possible biophysical mechanism for receptive fields.

It is intriguing that the frequency of phase synchronization in AM mode is of the order ∼0.1 Hz, in line with the low frequency Blood Oxygen Level-Dependent (BOLD) oscillations used to identify large scale brain networks and their properties from functional Magnetic Resonance Imaging (fMRI) (e.g. [108]). This finding supports the recent suggestion that these infra-slow oscillations are of astrocytic origin [109], [110]. Since the pattern of coordination observed here is based primarily on independent synaptic stimuli, one role of phase synchronization may be information encoding and communication [27].

### Extension of the AN model

Astrocytes, like neurons, also form interconnected networks where the communication between astrocytes is via gap junctions. Early research using cultures of hippocampal astrocytes showed that they were not only excitable by external stimulation but were also able to transmit or propagate intracellular Ca^2+^ oscillations to other non stimulated astrocytes [111]. This was followed by *in situ* experimentation which showed that glutamate released as a result of hippocampal neuronal activity results in both intracellular Ca^2+^ oscillations and intercellular waves (a wave which propagates through an astrocytic network) [6]. More recently, *in vivo* studies showed that astrocytes exhibit Ca^2+^ transients that travel much faster within the astrocyte syncytium than previously expected. Furthermore, Ca^2+^ transients have also been reported to be spontaneous and independent of neural activity [25], [111]–[114]. At present, however, it is still unclear if the Ca^2+^ waves are a result of external synaptic stimulation, coordinated spontaneous activity or a mixture of both [113], [114]. Whatever the source of intercellular Ca^2+^ waves, their ability to traverse relatively long distances suggests that coordination may not be solely local in nature, but may also be a means to realize flexible global communication across remote networks of neurons.

It is worth noting that the various model systems encompassed by the AN model were chosen to reduce computational overhead while still remaining biophysically meaningful. Thus, the AN model can form an important building block to explore global communication across large remote clusters of neurons via astrocyte ensembles. Extension of the AN model to include intercellular signaling similar to those described in [115]–[117] may aid in the understanding of how phase locking of field potentials encodes functionally relevant information via AN networks.

The present modeling results strengthen the hypothesis that astrocyte networks provide much more than structural support to neural networks. Indeed astrocytes are viewed as regulators of neural circuitry through coordination of transmission at synaptic junctions. Moreover, it is also believed that retrograde messengers induced in the postsynaptic neuron can be fed back either directly or via an astrocyte to receptors on the presynaptic neuron [106]. This feedback signal modulates the probability of neurotransmitter release and therefore may provide new insight into self repairing synapses Extension of the present AN model may thus be used to investigate such repair mechanisms [118]. Moreover, the AN model could be extended to provide a flexible research tool to allow neuroscientists to explore the role of astrocytes in a number of neurological disorders. For example, it has been suggested that a characteristic of Alzheimer's disease is the accumulation of amyloid-beta (Aβ) peptide, which can induce transient changes in intercellular Ca^2+^ concentration in astrocytes [119]. While this mechanism might explain the loss of new memory formation, further studies are required to understand the Aβ-induced neuronal and glia Ca^2+^ fluctuations and the changes that these fluctuations trigger.

## Supporting Information

Figure S1 **IP_3_ generation within the astrocyte cytoplasm.** (A) The evolution of IP_3_ within the cytoplasm of the astrocyte as a result of a range of Poisson generated spike trains stimulating the tripartite synapse. Note that IP_3_ builds much faster than it decays which can be seen after 10 s when the input ceases. (B) Same experiment as (A) except the Poisson distributed spike train is maintained. Note how the level of generated IP_3_ is limited to a steady state value and is dependent on the stimulus frequency.
(TIF)

Figure S2 **Examples of no Ca^2+^ oscillation.** (A) AM mode with input stimulus frequency set at 3 Hz. (B) FM mode with input stimulus set at 7 Hz. Note that in both cases the levels of IP_3_ are insufficient to cause a Ca^2+^ oscillation.
(TIF)

Figure S3 **Examples Ca^2+^ oscillations outside of the valid frequency range.** (A) AM mode with the input stimulus frequency set at 18 Hz. (B) FM mode with the input stimulus frequency set at 36 Hz. (C) AM-FM mode with the input stimulus frequency set at 11 Hz. In (A) AM mode this frequency causes Ca^2+^ to oscillate at a point at which it no longer crosses the threshold from above, therefore the gating function (* f *) remains active and in a state of depressing neurotransmitter release from the synapse. However, this negative feedback is insufficient to reduce the transmitter to which IP_3_ is degraded sufficiently to allow Ca^2+^ to drop below the threshold level. As a result IP_3_ reaches a steady state at which no further crossing of the threshold is possible. This is also the case with (B) and (C); however, in these cases the steady state level of IP_3_ now causes a cessation of the Ca^2+^ oscillation also.
(TIF)
