# Supplementary figures and images for: Bidirectional Coupling between Astrocytes and Neurons Mediates Learning and Dynamic Coordination in the Brain: A Multiple Modeling Approach
Source: PLoS One. 2011 Dec 29;6(12):e29445. doi: 10.1371/journal.pone.0029445 (PMC3248449; doi:10.1371/journal.pone.0029445)

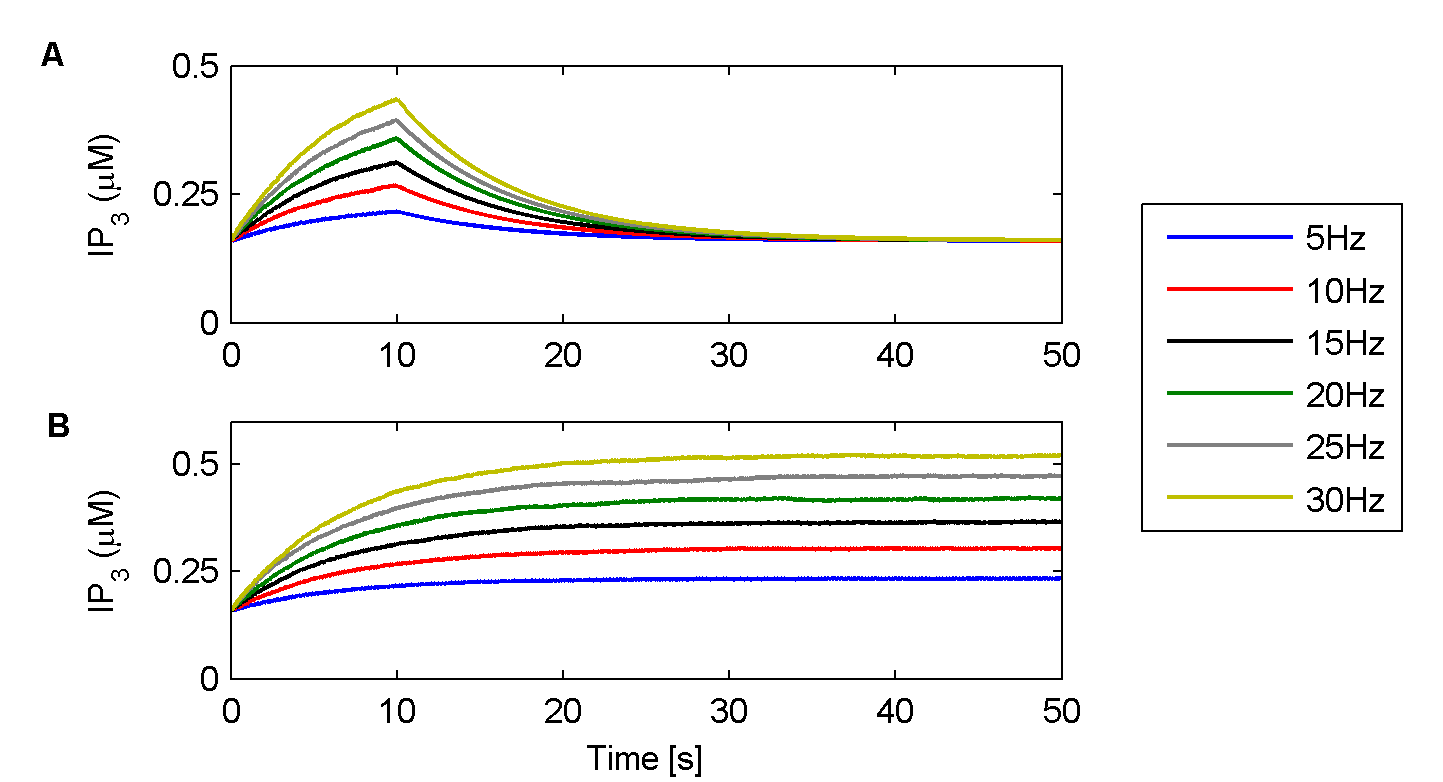

Supplement: Figure S1 — IP3 generation within the astrocyte cytoplasm. (A) The evolution of IP3 within the cytoplasm of the astrocyte as a result of a range of Poisson generated spike trains stimulating the tripartite synapse. Note that IP3 builds much faster than it decays which can be seen after 10 s when the input ceases. (B) Same experiment as (A) except the Poisson distributed spike train is maintained. Note how the level of generated IP3 is limited to a steady state value and is dependent on the stimulus frequency. (TIF) [file pone.0029445.s001.tif]

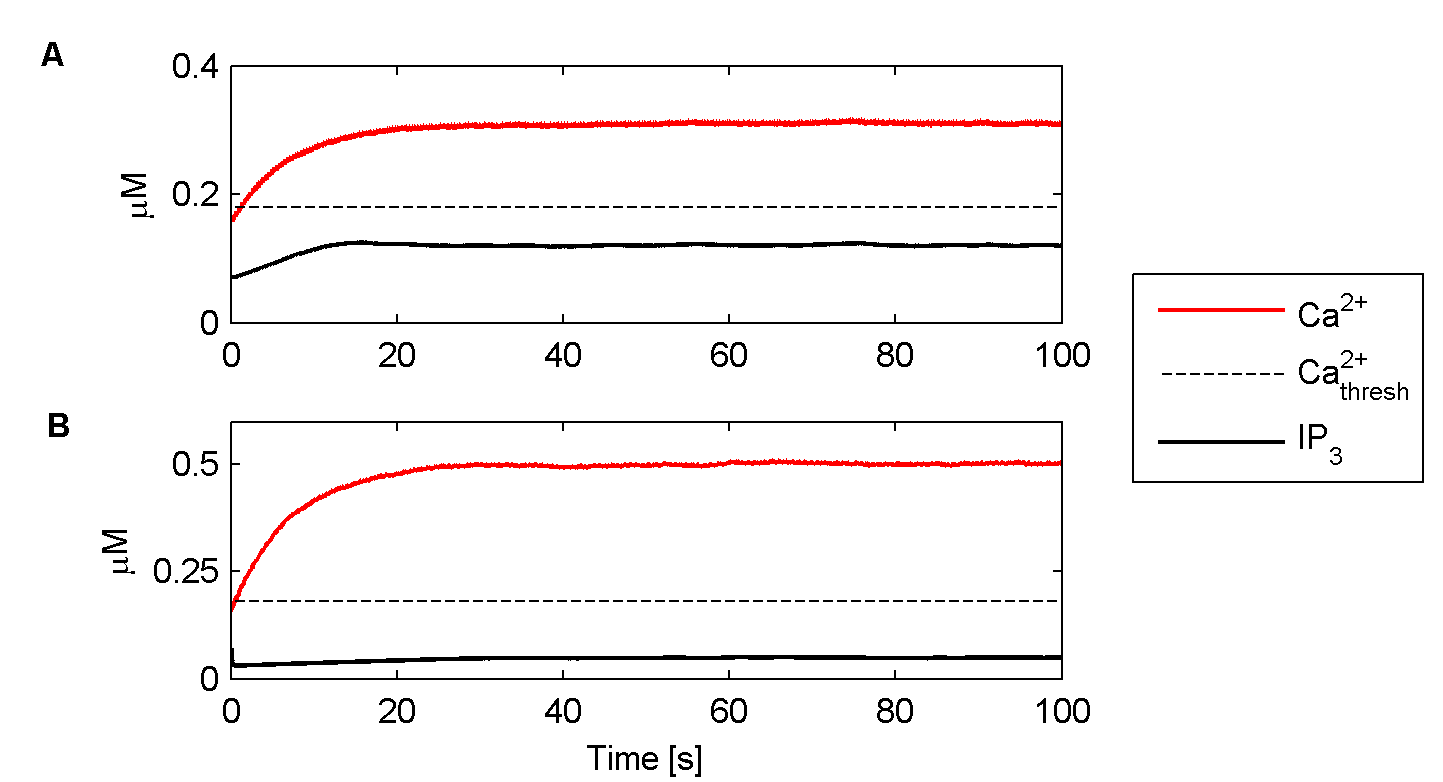

Supplement: Figure S2 — Examples of no Ca2+ oscillation. (A) AM mode with input stimulus frequency set at 3 Hz. (B) FM mode with input stimulus set at 7 Hz. Note that in both cases the levels of IP3 are insufficient to cause a Ca2+ oscillation. (TIF) [file pone.0029445.s002.tif]

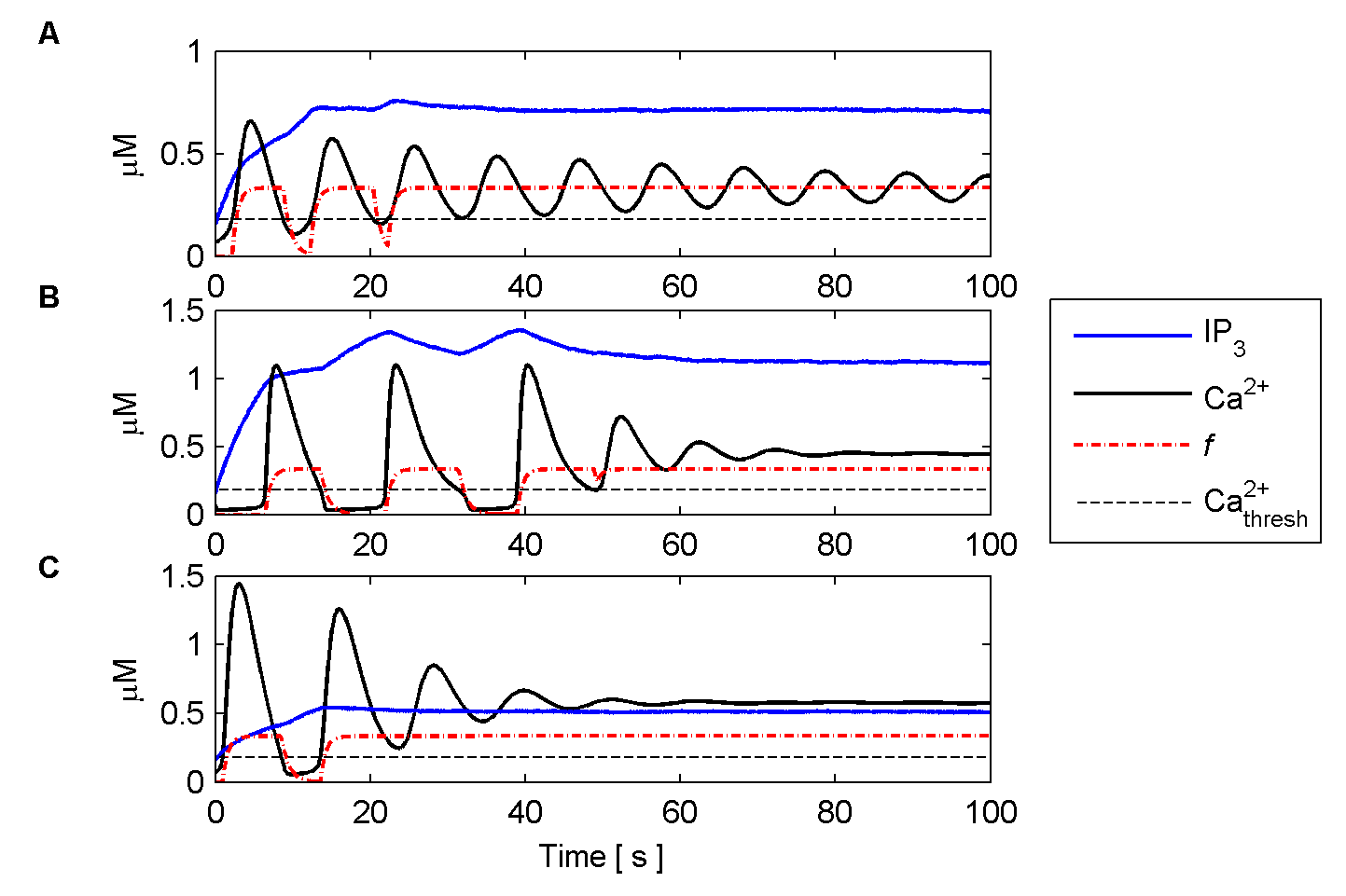

Supplement: Figure S3 — Examples Ca2+ oscillations outside of the valid frequency range. (A) AM mode with the input stimulus frequency set at 18 Hz. (B) FM mode with the input stimulus frequency set at 36 Hz. (C) AM-FM mode with the input stimulus frequency set at 11 Hz. In (A) AM mode this frequency causes Ca2+ to oscillate at a point at which it no longer crosses the threshold from above, therefore the gating function ( f ) remains active and in a state of depressing neurotransmitter release from the synapse. However, this negative feedback is insufficient to reduce the transmitter to which IP3 is degraded sufficiently to allow Ca2+ to drop below the threshold level. As a result IP3 reaches a steady state at which no further crossing of the threshold is possible. This is also the case with (B) and (C); however, in these cases the steady state level of IP3 now causes a cessation of the Ca2+ oscillation also. (TIF) [file pone.0029445.s003.tif]
